# Community transmission of mpox clade Ib not driven through sexual exposures, Uvira, eastern Democratic Republic of the Congo, June to October 2024

**DOI:** 10.2807/1560-7917.ES.2025.30.50.2500280

**Published:** 2025-12-18

**Authors:** Patrick Musole Bugeme, Patrick Kazuba Bugale, Trust Faraja Mukika, Megan O’Driscoll, Javier Perez-Saez, Levi Bugwaja, Salomon Mashupe Shangula, Willy Kasi, Justin Bengehya, Stephanie Ngai, Antonio Isidro Carrion Martin, Jules Jackson, Patrick Katoto, Esto Bahizire, Noella Mulopo-Mukanya, Justin Lessler, Jackie Knee, Pauline Vetter, Elizabeth C Lee, Daniel Mukadi-Bamuleka, Andrew S Azman, Espoir Bwenge Malembaka

**Affiliations:** 1 Department of Epidemiology, Johns Hopkins University, Baltimore, United States; 2 Center for Tropical Diseases and Global Health (CTDGH), Université Catholique de Bukavu, Bukavu, Democratic Republic of the Congo; 3 Hôpital Général de Référence d’Uvira, Uvira Health Zone, Uvira, Democratic Republic of the Congo; 4 Institute of Global Health, University of Geneva, Geneva, Switzerland; 5 Geneva Centre for Emerging Viral Diseases, Geneva University Hospitals, Geneva, Switzerland; 6 Division Provinciale de la Santé Publique du Sud-Kivu, Bukavu, Democratic Republic of the Congo; 7 Médecins Sans Frontières, Uvira, Democratic Republic of the Congo; 8 Médecins Sans Frontières, London, United Kingdom; 9 Centre de Recherche en Sciences Naturelles de Lwiro, Bukavu, Democratic Republic of the Congo; 10 Rodolphe Merieux INRB-Goma Laboratory, Goma, Democratic Republic of the Congo; 11 Department of Epidemiology, Gillings School of Global Public Health, University of North Carolina at Chapel Hill, Chapel Hill, United States; 12 Carolina Population Center, University of North Carolina at Chapel Hill, Chapel Hill, United States; 13 Department of Disease Control, London School of Hygiene & Tropical Medicine, London, United Kingdom; 14 Geneva Centre of Humanitarian Studies, Faculty of Medicine, University of Geneva, Geneva, Switzerland; 15 Department of Virology, Institut National de Recherche Biomédicale (INRB), Kinshasa, Democratic Republic of the Congo; 16 Service de Microbiologie, Département de Biologie Médicale, Cliniques Universitaires de Kinshasa, Université de Kinshasa, Kinshasa, Democratic Republic of the Congo; 17 Division of Tropical and Humanitarian Medicine, Geneva University Hospitals (HUG), Geneva, Switzerland

**Keywords:** Mpox, clade Ib, sexual transmission, age distribution, mortality, pregnancy, malnutrition, DR Congo

## Abstract

**BACKGROUND:**

In September 2023, monkeypox virus (MPXV) clade Ib emerged in Kamituga, a mining zone in South Kivu, Democratic Republic of the Congo (DRC), primarily through sexual transmission.

**AIM:**

We aimed to investigate cases in a MPXV clade Ib outbreak in Uvira, eastern DRC.

**METHODS:**

From June to October 2024, we collected demographic, exposure and clinical data from suspected mpox cases at Uvira hospital and in households. The virus was identified by PCR. We investigated putative transmission patterns, disease severity and risk factors.

**RESULTS:**

We identified 973 suspected cases: 415 (42.7%) were tested with PCR and 322 (77.6%) were confirmed. The median age of suspected cases was 9 years (interquartile range (IQR): 3–20 years), with 620 (63.7%) aged < 15 and 344 (35.4%) < 5 years. Severe disease (≥ 100 lesions) was more common in cases aged < 15 years (25.6%; 142/554) than others (16.1%; 49/304; p < 0.001). Twenty-two (12.2%) of 181 cases aged < 5 years had acute malnutrition. Seven cases died; the overall case-fatality ratio was 0.7%, and in infants (aged < 1 year) it was 3.9% (5/127). Of 329 suspected cases tested for HIV, six (1.8%) were positive. Nineteen (14.5%) of 131 females aged 15–49 years were pregnant. Most reported exposures to suspected mpox cases occurred in households (67.9%; 298/439). Sexual (6.0%; 19/318) or healthcare-related occupational exposures (1.4%; 6/417) were less common. Animal exposures were few (5.0%; 39/776) and predominantly domestic (97.4%; 38/39).

**CONCLUSION:**

This child-centred outbreak, driven by non-sexual transmission, underscores the need for paediatric vaccines, nutritional support and household interventions. Adult-focused responses alone may be insufficient to control the outbreak.

**Figure:**
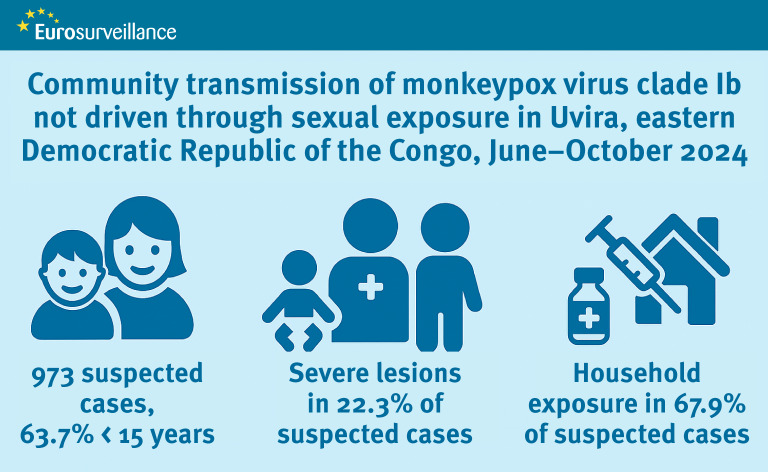

**Key public health message**

**What did you want to address in this study and why?**

Mpox, caused by monkeypox virus (MPXV), is a disease presenting with fever and skin rash. A new variant of MPXV, called clade Ib, appeared in Kamituga, Democratic Republic of the Congo in September 2023, mainly spreading through heterosexual contact in mining communities. By mid-2024, an outbreak occurred in Uvira, 380 km away, mostly affecting children. This study offers new insights into the spread, symptoms and vulnerability to MPXV clade Ib in Uvira.

**What have we learnt from this study?**

In Uvira, children under 15 years were most affected (620/973; 63.7%) by the clade Ib outbreak, and few cases were linked to sexual contact. Healthcare- and work-related exposures were rare among cases (1.4% and 1.6%, respectively). While the overall death rate was low (0.7%), it was 5.6 times higher (3.9%) in infants. This pattern differs from outbreaks among adults linked to sexual exposures in other areas.

**What are the public health implications?**

Monkeypox virus has been spreading in crowded homes, especially in areas with many children and limited healthcare. To stop the virus and protect the most vulnerable, speeding up the development of vaccines for children and supporting prevention efforts in homes and communities are needed.

## Introduction

Monkeypox virus (MPXV) has been documented in humans in the Democratic Republic of the Congo (DRC) since 1970, historically causing small-scale zoonotic mpox outbreaks and primarily affecting young children in the western and central provinces. These outbreaks were characterised by limited person-to-person transmission, with no considerable differences in infection rates by sex [1-3].

Two distinct MPXV clades (clade I and II) with variable geographic distribution are circulating in the DRC, each comprising two subclades (a and b), and differing case-fatality ratios (CFR) influenced by factors such as clade virulence, host vulnerability, comorbidities and access to care. Clade Ia, predominant in Central Africa and presumed more virulent, has a CFR reaching 10% [4,5]. Clade IIa is more common west of the Dahomey Gap, from Ghana to Sierra Leone, with a CFR below 0.1%, while clade IIb predominates east of the Dahomey Gap in Nigeria and shows more variable CFRs, though generally lower than clade Ia [6,7]. In September 2023, the South Kivu province of the DRC reported its first-ever mpox outbreak in the remote mining region of Kamituga, caused by a newly identified virus variant, clade Ib [8]. The virus rapidly spread across the Kivu provinces, Burundi, Rwanda, Uganda, Kenya and beyond. This, combined with increased transmission of other MPXV variants, mainly within the DRC but also in other African settings, prompted World Health Organization (WHO) to declare mpox a public health emergency of international concern in August 2024 [9-11].

Reports from Kamituga, where the clade Ib outbreak was first identified and where most patients were adults, suggested that sexual contact was the primary mode of transmission, and earlier studies in western DRC had already linked MPXV clade I infections to sexual exposure [12]. A cohort study conducted between May and October 2024 at the Kamituga Mpox Treatment Centre (MTC) found that 75% of all 510 suspected mpox cases and 79% of 407 confirmed cases were aged ≥ 15 years [13]. Notably, genital lesions were nearly three times more prevalent in patients aged ≥ 15 years (89%) than among children aged < 5 years (32%). Furthermore, 40% of patients with available contact information reported sexual exposure to a case. Another study conducted in the same area showed that nearly nine of 10 patients reported recent sexual contact in bars or hotels [14]. However, official surveillance data suggested that as the outbreak spread to other health zones of South Kivu, the proportion of cases among children has increased, indicating a possible shift towards non-sexual transmission pathways in these areas [15]. The overall CFR of clade Ib infections in Kamituga was relatively low, at 0.1%, but notably higher among children < 5 years, reaching 6.7% [16].

The first mpox cases in Uvira, situated in South Kivu, approximately 380 km from Kamituga, were officially reported on 2 May 2024, 8 months after the outbreak began in Kamituga. An MTC was opened at the Uvira General Hospital on 8 June 2024. Since 17 June 2024, a free mpox care package has been provided at the MTC with support from Médecins Sans Frontières, covering supportive treatment for mpox and related complications, essential medications, and meals for patients and caregivers.

Here, we provide new insights into the epidemiology, clinical features and risk factors associated with MPXV clade Ib in Uvira.

## Methods

### Setting, design and population

We conducted a descriptive surveillance study of mpox in Uvira, the DRC, a trading city on the northern shores of Lake Tanganyika, from 3 June to 24 October 2024. Uvira health zone is a mixture of rural and urban areas sharing land border with Burundi and connected to Tanzania and Zambia through the DRC’s second largest port. The city includes 18 of the 22 health areas of the Uvira health zone. Approximately 65% of the 460,000 people in the Uvira health zone live in the city (City of Uvira Official Population Count Data, 2024), with an estimated population density of 17,988 people per km^2^. The median household size in Uvira city is eight individuals [17,18]. Agriculture, fisheries and trading play an important part in the local economy [19].

As of April 2024, South Kivu province hosted around 1.9 million of the 7 million people internally displaced in eastern DRC [20], exact official numbers at health zone level are not publicly available. Uvira also hosts refugees, mainly from Burundi, due to ongoing conflicts in the region. The city is highly vulnerable to climate-related disasters [20,21].

### Clinical case definition and data collection

At the Uvira MTC, anyone presenting with a rash or skin lesions during the study period was considered a suspected mpox case and eligible for this analysis. A detailed description of the case definition is presented in Supplementary File. Suspected case basic data were recorded in MTC’s paper triage register, including patient identifiers, sex, age, profession, home address (health area and avenue), date of hospital admission or outpatient visit, symptom onset date, presence of fever, skin rash or lesions on palms or soles, sample collection status, MPXV laboratory result, hospitalisation or outpatient management status, smallpox vaccination status, outcome details (dead, discharged, transferred) and date of outcome.

Two health zone physicians conducted clinical examinations and administered an in-depth electronic form via the KoboCollect platform (https://www.kobotoolbox.org), following WHO mpox surveillance guidelines [22]. This form captured all the paper register data along with additional details on clinical signs, symptoms and medical history, exposures and travel within the past 21 days, and care-seeking behaviour. Vulnerability data, including pregnancy, breastfeeding and child nutritional status, assessed using standard mid-upper arm circumference (MUAC) measurements [23], were collected. A discharge form was also completed for each patient to record clinical outcomes. Where residential addresses were available and located within the city, a team of three nurses visited the households of cases residing in the city to gather basic information on household composition and characteristics.

### Sample collection and testing

Specimens from skin lesions were collected with a dry swab by trained MTC staff. When not possible, oropharyngeal samples were collected in a viral transport medium. Samples were maintained at 4°C and transported to either the Rodolphe Mérieux INRB Goma Laboratory or the South Kivu Provincial Laboratory in Bukavu for testing, as presented in Supplementary File.

At the Rodolphe Mérieux INRB Goma Laboratory, samples were tested by qPCR with the Radi Fast Mpox Detection Kit (KH Medical, Pyeongtaek-si, South Korea) to detect MPXV clades I and/or II. We considered a quantification cycle (Cq) value of < 40 threshold as positive. Additionally, a subset of samples was clade-typed with LightMix Modular Monkeypox (Roche, Basel, Switzerland) [24].

At the South Kivu Provincial Laboratory, samples were analysed using the GeneXpert platform (Xpert; Mpox, Cepheid, Sunnyvale, the United States (US)), designed to detect DNA from MPXV clade II and non-variola orthopoxvirus (OPX) genes in human specimens. Samples were classified as positive for MPXV clade I when non-variola orthopoxvirus DNA was detected with no amplification of the MPXV clade II target, following the manufacturer’s instructions [25].

Whenever rapid diagnostic tests (RDTs) were available, consenting hospitalised patients were screened for HIV and syphilis using the SD Bioline HIV/Syphilis Duo test (Abbott Diagnostics, Lake County, US). For patients testing positive for HIV on this RDT, two additional tests were performed with HIV 1/2 Stat-Pak Assay (Chembio Diagnostic Systems, Medford, US) and Uni-Gold HIV (Trinity Biotech, Bray, Ireland) as per the DRC’s HIV control programme algorithm.

### Data management and analysis

We calculated the weekly number of clinical cases recorded at the Uvira MTC, by age group (< 1 year, 1–4 years, 5–14 years, 15–44 years and ≥ 45 years) and laboratory confirmation. We used WHO guidance to classify disease severity as mild (< 25 skin lesions), moderate (25–99 lesions), severe (100–250 lesions) and very severe or grave (> 250 lesions) [26]. For children aged 3–59 months with MUAC measurements, we calculated MUAC-for-age (MFA) z-scores using the *zscorer* package in R [27]. Nutritional status was classified as normal (MFA ≥ −2), moderate acute malnutrition (−3 ≤ MFA < −2) or severe acute malnutrition (MFA < −3) [23,28].

We used data from four population-representative surveys conducted in Uvira city between 2021 and 2024 [18] (n = 10,604) to estimate the demographic structure of the population given that no census has been conducted in the DRC since 1984. These data allowed us to compare the age distribution of suspected mpox cases with that of the general Uvira population. We also compared the age distribution of cases with that of their households, based on age composition collected during home visits.

For exposure assessment, we first described the patients who reported at least one exposure to a suspected mpox case. We then summarised the exposure types, treating each reported exposure as an independent event for individuals reporting multiple exposures. The characteristics of the study participants were compared using the Wilcoxon rank sum, Pearson's chi-square or Fisher's exact tests. All analyses were performed using R version 4.4.1 (https://www.r-project.org/).

## Results

### Socio-demographic and household characteristics

Between 3 June and 24 October 2024, 973 suspected mpox cases were recorded at the Uvira MTC, with 50.5% (n = 491) being female. The median age of patients was 9 years (interquartile range (IQR): 3–20 years). Most cases were children and adolescents, with 63.7% (n = 620) aged < 15 years (Table 1) and 35.4% (n = 344) < 5 years (Figure). More detailed information is presented in Supplementary Tables S1 and S3. The proportion of cases aged < 15 years increased as the outbreak progressed until mid-July 2024 (Figure). The suspected mpox cases were younger than the overall Uvira population, with those aged < 5 years being particularly overrepresented (Figure). Patients’ median household size was eight (IQR: 5–10) people, with a median of four (IQR: 2.7–5.4) people per sleeping room (Table 1). Female sex workers made up 1.2% (n = 12) of the total cases, and only four (0.4%) cases were healthcare workers (Table 1).

**Table 1:** Socio-demographic characteristics and exposure history of suspected mpox cases seeking healthcare at the Uvira Mpox Treatment Centre, Democratic Republic of the Congo, 3 June–24 October 2024 (n = 973)^a^

Characteristic	Overall (n = 973)		< 15 years (n = 620)		≥ 15 years (n = 353)		p value
	n	%	n	%	n	%	
Age (years)							
Median	9		4		24		NA
Interquartile range	3.0–20.0		1.8–8.0		19.0–31.0		
Sex							
Female	491	50.5	302	48.7	189	53.5	0.147
Male	482	49.5	318	51.3	164	46.5	
Household size^b^							
Median number of people	8		8		7		0.104
Interquartile range	5.0–10.0		6.0–10.0		5.0–10.0		
Median number of people per sleeping room in the household	4		4		3		< 0.001
Interquartile range	2.5–5.0		3.0–5.0		2.0–5.0		
Occupation							
Student	310	31.9	245	39.5	65	18.4	NA
Preschool age children	293	30.1	293	47.3	0	0	
Small business	83	8.5	7	1.1	76	21.5	
Farmer	18	1.8	6	1.0	12	3.4	
Female sex worker	12	1.2	0	0	12	3.4	
Educator/Teacher	9	0.9	0	0	9	2.6	
Driver	12	1.2	0	0	12	3.4	
Artisan (manual work)	9	0.9	1	0.2	8	2.3	
Cyclist porter	7	0.7	0	0	7	2.0	
Healthcare worker	4	0.4	0	0	4	1.1	
Working in mining site	4	0.4	2	0.3	2	0.6	
No occupation	69	7.1	6	1.0	63	17.8	
Number of sexual partners 21 days before symptom onset (n = 286)^c^							
0	84	29.4	NA		84	29.4	NA
1	153	53.5			153	53.5	
2–5	42	14.7			42	14.7	
≥ 6	7	2.4			7	2.4	
People reporting contact with at least one suspected mpox case (n = 743)^d^							
Yes	346	46.6	236/474	49.8	110/269	40.9	0.019
Number of contacts with a suspected mpox case (n = 346)^d^							
1	276	79.8	182/236	77.1	94/110	85.5	0.065
2	43	12.4	36/236	15.3	7/110	6.4	
≥ 3	27	7.8	18/236	7.6	9/110	8.2	
Sexual exposure to a suspected mpox case (n = 318)^c^							
Yes	19	6.0	0/214	0	19/104	18.3	< 0.001
Animal contact (n = 776)							
Yes	39	5.0	28/508	5.5	11/268	4.1	0.394
Type of animal contact (n = 39)							
Domestic animals	38	97.4	28/28	100.0	10/11	90.9	0.282
Wild animals	1	2.6	0/28	0	1/11	9.1	
Travel (n = 845)^d^							
Travelled outside Uvira	90	10.7	17/547	3.1	73/298	24.5	< 0.001
International travel (n = 31)^d^							
Burundi	30	96.8	2/2	100.0	28/29	96.6	> 0.999
Tanzania	1	3.2	0/2	0	1/29	3.4	

NA: not applicable.

^a^ Suspected cases reported being exposed to at least one suspected mpox case are included in the table.

^b^ Household characteristics data were not available for non-residents of Uvira city and Uvira residents for whom a household visit was not conducted mainly due to lack of accurate residential address.

^c^ Sexual exposure was reported only for individuals aged ≥ 15 years who were considered likely to be sexually active. Occupation was a multiple-choice question.

^d^ All questions about exposure and travel history referred to the 3 weeks preceding the onset of fever or skin rash.

**Figure:**
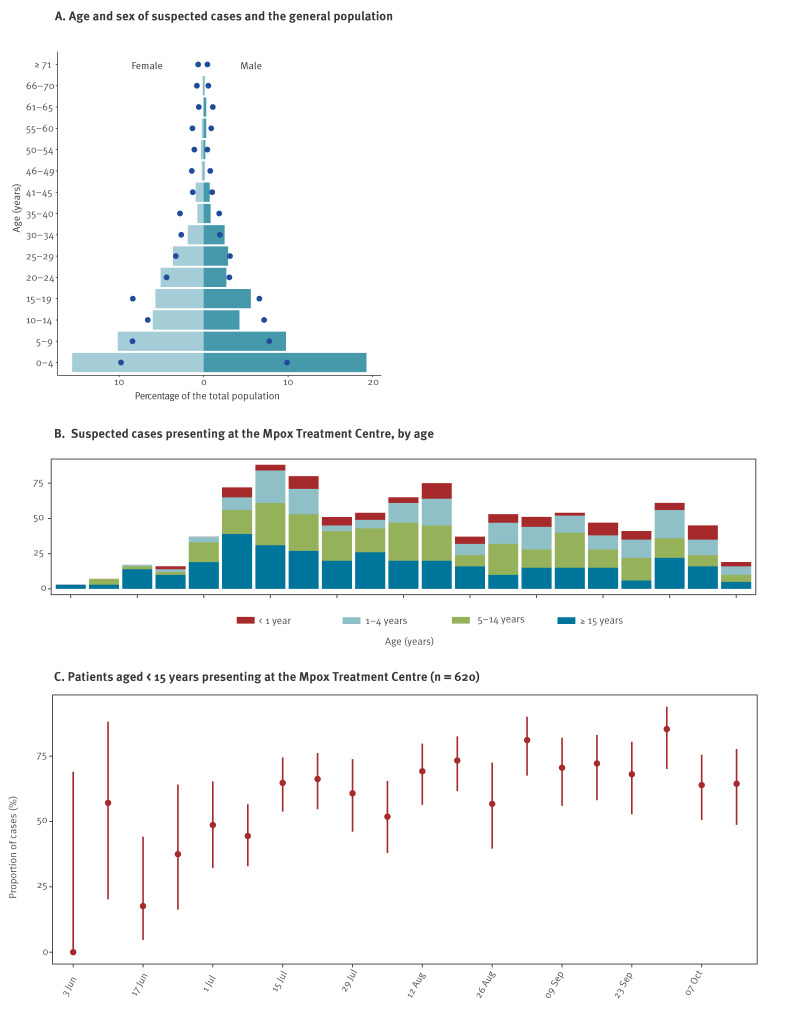
Age and sex distribution of suspected mpox cases seeking healthcare at the Uvira Mpox Treatment Centre, Democratic Republic of the Congo, 3 June–24 October 2024 (n = 973)

Panel A. Sex distribution of the cases: females (n = 491) and males (n = 482). Age-sex pyramid of suspected cases and the general population of Uvira. The blue dots denote the disaggregation by age and sex from the population estimates based on household composition data from four cross-sectional surveys conducted in Uvira between 2021 and 2024 (n = 10,604 household members).

Panel B. Epidemic curve by age group and week of presentation at the Mpox Treatment Centre.

Panel C. Weekly proportion depicted.

### Clinical characteristics

While all suspected cases were meant to have a sample collected and tested, only a subset had test results available due to laboratory supply and logistical limitations. Of the 973 suspected cases, 415 (42.7%) were tested by at least one assay, with 322 (77.6%) cases confirmed by PCR (Table 2). More details of the patients tested are presented in Supplementary Tables S3 and S4. Of the 49 positive samples tested with the clade-typing assay, all were confirmed as clade Ib. Patients with laboratory test results had similar socio-demographic characteristics as those without test results but had more symptoms and lesions, as explained in Supplementary Table S3. Patients testing positive had socio-demographic and clinical characteristics comparable to those with negative results, as presented in Supplementary Table S4.

**Table 2:** Clinical characteristics of suspected mpox cases, by age, on admission to Uvira Mpox Treatment Centre, the Democratic Republic of Congo, 3 June–24 October 2024 (n = 973)

Characteristic	Overall (n = 973)		< 15 years (n = 620)		≥ 15 years (n = 353)		p value
	n	%	n	%	n	%	
Hospitalisation (n = 860)							
Yes	431	50.1	273/554	49.3	158/306	51.6	0.508
Clinical sign							
Self-reported fever	648/836	77.5	426/536	79.5	222/300	74.0	0.069
Lesions on palms and soles	320/872	36.7	200/561	35.7	120/311	38.6	0.389
Cervical adenopathy	550/870	63.2	400/559	71.6	150/311	48.2	< 0.001
Inguinal adenopathy	664/872	76.1	422/561	75.2	242/311	77.8	0.390
Axillary adenopathy	288/867	33.2	214/557	38.4	74/310	23.9	< 0.001
Generalised adenopathy	205/840	24.4	160/538	29.7	45/302	14.9	< 0.001
Eye redness	113/861	13.1	69/554	12.5	44/307	14.3	0.435
Eye itching	74/513	14.4	33/209	15.8	41/304	13.5	0.466
Light sensitivity	84/489	17.2	36/193	18.7	48/296	16.2	0.485
Oral lesions	204/852	23.9	135/549	24.6	69/303	22.8	0.552
Genital lesions	413/860	48.0	217/554	39.2	196/306	64.1	< 0.001
Anal lesions	123/852	14.4	73/548	13.3	50/304	16.4	0.214
Number of lesions (n = 858)							
< 25	238	27.7	123/554	22.2	115/304	37.8	< 0.001
25–99	429	50.0	289/554	52.2	140/304	46.1	
100–250	125	14.6	102/554	18.4	23/304	7.6	
> 250	66	7.7	40/554	7.2	26/304	8.6	
Pregnancy and breastfeeding							
Pregnant^a^	19/131	14.5	NA		19/131	14.5	NA
Breastfeeding mother^a^	27/120	22.5			27/120	22.5	
Breastfed child^b^	128/166	77.1	128/166	77.1	NA		
Nutritional status of children aged 3–59 months (n = 181)							
Severe acute malnutrition	10	5.5	10	5.5	NA		
Moderate acute malnutrition	12	6.6	12	6.6			
Normal	159	87.8	159	87.8			
Syphilis test result (n = 273)							
Negative	272	99.6	171/171	100.0	101/102	99.0	0.374
Positive	1	0.4	0/171	0.0	1/102	1.0	
HIV test result (n = 329)							
Negative	323	98.2	214/214	100.0	109/115	94.8	0.002
Positive	6	1.8	0/214	0.0	6/115	5.2	
Mpox confirmation (PCR) (n = 415)							
Negative	93	22.4	68/264	25.8	25/151	16.6	0.031
Positive	322	77.6	196/264	74.2	126/151	83.4	

NA: not applicable.

^a^ Questions about pregnancy and breastfeeding were only asked to female patients aged 15–49 years (pregnancy: (n = 131) and breastfeeding: (n = 120)).

^b^ Child’s breastfeeding status was only asked for those aged ≤ 2 years (n = 166).

Severe to very severe mpox (≥ 100 lesions) was observed in 22.3% (n = 191/858) of suspected cases, with a higher proportion in children and adolescents aged < 15 years (25.6%; 142/554) compared with older individuals (16.1%; 49/304; p < 0.001) (Table 2). A similar finding was seen in confirmed cases, where 31.6% (61/193) of children and adolescents aged < 15 years had severe to very severe mpox, vs 17.1% (20/117) of individuals aged ≥ 15 years, as presented in Supplementary Table S5. Genital lesions were observed in 48.0% (413/860) of suspected cases, significantly more frequently in individuals aged ≥ 15 years (64.1%; 196/306) than in younger patients (39.2%; 217/554; p < 0.001). This pattern was even more marked in confirmed cases, where 75.2% (88/117) of patients aged ≥ 15 years had genital lesions compared with 45.6% (88/193) of younger patients (p < 0.001), as presented in Supplementary Table S5. Anal lesions were reported in 14.4% (123/852) of suspected cases and 17.6% (54/306) of confirmed cases, with no significant differences by age or sex, as presented in (Table 2) and Supplementary Table S5. Cervical, axillary and generalised lymphadenopathy was more common in patients aged < 15 years than in older individuals, in both suspected and confirmed cases (p < 0.001).

Among 329 patients who consented to HIV testing, six (1.8%) had a positive test result, all of whom were aged ≥ 15 years (5.2% age group positivity, 6/115), and with no significant difference by sex (Table 2). More details are presented in Supplementary Table S2. One (0.4%) case with syphilis was detected among the 273 tested cases.

Of 131 female patients of reproductive age (15–49 years), 14.5% (n = 19) self-reported as pregnant, with seven of 19 in their third trimester, as presented in Supplementary Table S6, and three presenting with severe to very severe suspected mpox. Follow-up data were available for nine pregnant patients. Among these nine, four had a positive PCR test, one had a negative PCR and four with no laboratory results. They delivered nine live newborns (including one set of twins), with no complications or lesions reported by mothers, and one stillbirth, which occurred in a person who tested positive for MPXV and was in the first trimester at the time of admission to the MTC. Two additional pregnant persons followed up were still pregnant at the time of follow-up with no complication reported. Among 181 children aged < 5 years with MUAC measurements, 22 (12.1%) had acute malnutrition, including 10 (5.5%) children with severe and 12 (6.6%) with moderate malnutrition (Table 2).

Seven cases at the MTC died during the study period, with ages ranging between 13 days and 32 years, as presented in Supplementary File. The overall CFR was 0.7% (95% confidence interval (CI): 0.4–1.5) among all suspected cases. Time from hospitalisation to death varied from 3 to 29 days. Six of the seven patients who died had PCR results: five tested positive and one tested negative. Five infants (aged < 1 year) died, including three newborns (aged < 1 month), yielding a CFR of 3.9% (5/127; 95% CI: 1.7–8.9) in this age group. Two adults with weakened immune systems died: both with HIV, one presented with disseminated necrotising lesions (described elsewhere [29]), and the other with co-existing diabetes. Two of the six cases that tested positive for HIV died. Detailed clinical descriptions of the deaths are provided in Supplementary Table S7.

### Mpox exposures

Exposure data were collected for the 21-day-period preceding symptom onset. Direct contact with a suspected mpox case was reported by 46.6% (346/743) patients: 79.8% (n = 276) were exposed to a single case and 20.2% (n = 70) to multiple suspected cases (Table 1), with no significant difference by sex. Ninety (10.7%) of 845 patients reported travelling: 31 (34.4%) travelled internationally, 30 of them to neighbouring Burundi. Only 5.0% (39/776) of patients reported contact with animals, most contacts involved domestic animals (n = 38). No contact with rodents was reported. Healthcare occupational exposures accounted for 1.4% (6/417) of all exposures (Table 3).

**Table 3:** Description of exposure events of suspected mpox cases seeking healthcare at the Uvira Mpox Treatment Centre, Democratic Republic of the Congo, 3 June–24 October 2024 (n = 460)^a^

Characteristic	Overall (n = 460)			< 15 years (n = 321)			≥ 15 years (n = 139)		p value
	n	%		n		%	n	%	
Sex									
Male	239	52.0		185	57.6		54	38.8	< 0.001
Female	221	48.0		136	42.4		85	61.2	
Number of contacts (n = 401)									
1	28	7.0		13/279	4.7		15/122	12.3	0.006
≥ 2	373	93.0		266/279	95.3		107/122	87.7	
Relationship with a suspected case (n = 417)									
Child	30	7.2		0/287	0.0		30/130	23.1	NA
Parent	13	3.1		11/287	3.8		2/130	1.5	
Spouse	11	2.6		0/287	0.0		11/130	8.5	
Sex partner	8	1.9		0/287	0.0		8/130	6.2	
Another household member (e.g. sibling)	197	47.2		161/287	56.1		36/130	27.7	
Another non-household relative, friend, colleague, neighbour	152	36.5		112/287	39.0		40/130	30.8	
Healthcare occupational exposure	6	1.4		3/287	1.0		3/130	2.3	
Exposure (n = 439)									
Contact in Uvira	423		96.4	298/304	98.0		125/135	92.6	0.010
Exposure at restaurants, bars, hotels, nightclubs	108		24.6	75/304	24.7		33/135	24.4	0.959
Household exposure	298		67.9	208/304	68.4		90/135	66.7	0.716
Exposure at workplace	7		1.6	0/304	0.0		7/135	5.2	< 0.001
Exposure at school	3		0.7	3/304	1.0		0/135	0.0	0.556
Exposure at sport place	1		0.2	1/304	0.3		0/135	0.0	> 0.999
Exposure at healthcare facility	14		3.2	6/304	2.0		8/135	5.9	0.039
Hospitalisation of the reported contact person (n = 231)									
Yes	191		82.7	145/169	85.8		46/62	74.2	0.039

NA: not applicable.

^a^ All questions refer to the 21-day period preceding symptom onset. Contact history with suspected mpox cases was available for 346 patients, some of whom reported exposure to multiple suspected cases or multiple locations. For individuals reporting multiple exposures, each exposure was counted as a separate event.

Overall, 70.5% (202/286) of cases aged ≥ 15 years reported having at least one sexual partner in the 3 weeks before symptom onset, and 17.1% (49/286) reported multiple sexual partners (Table 1). Nineteen (6.0%) of 318 participants reported sexual exposure to a suspected case.

Reported exposures to suspected mpox were primarily from within case households (67.9%; 298/439) (Table 3), and among these, 62.7% (187/298) involved exposure to multiple suspected cases. Of 439 respondents, 3.2% (n = 14) reported exposure within a healthcare facility. Exposures were also reported in restaurants, bars, hotels and nightclubs (24.6%, 108/439).

## Discussion

The MPXV clade Ib outbreak in Uvira primarily affected children and adolescents aged < 15 years (63.7%), with limited reported sexual transmission. Occupational healthcare and workplace exposures were rare (1.4% and 1.6%, respectively). The overall CFR was 0.7% but higher in infants.

Several factors likely contributed to the high incidence of MPXV clade Ib among children: (i) cryptic transmission; (ii) prior smallpox immunity in older individuals; and (iii) age-related differences in MPXV susceptibility, exacerbated by child malnutrition. Remarkably, the age distribution of clade Ib cases in Uvira mirrors patterns from historical clade Ia outbreaks [2,30]. However, despite higher mortality among young children, the CFR for clade Ib in infants was lower than the 10% CFR reported in paediatric populations during clade Ia outbreaks [5,31]. This lower CFR may reflect the reduced virulence of clade Ib, possibly due to OPG032 gene deletion, which encodes the complement control protein, a key virulence factor [32,33]. Additionally, in remote and hard-to-reach areas where most historical clade Ia outbreaks occurred, surveillance bias may have led to an overestimation of CFR, as only the most severe cases would seek care, with milder cases likely remaining at home. Finally, improved and facilitated healthcare access in Uvira may have prevented deaths. Unlike previous outbreaks in remote and hard-to-reach areas, this one received international support, with non-governmental organisations and the Ministry of Health providing free treatment, food and hygiene kits.

This outbreak reflects a shift from sexual to predominantly non-sexual transmission, with overcrowded housing likely accelerating the spread. Thus, in Uvira where the median household size was eight and a median of four people slept in the same room, the home isolation guidance of WHO is difficult to follow, a situation common to many similar settings. While isolation at the MTC can reduce household spread, it imposes indirect costs on families and strains on the health system. Despite the observed shift towards non-sexual transmission, some degree of sexual transmission likely persists, particularly among individuals aged ≥ 15 years, who had a higher frequency of genital lesions compared with younger patients. These complexities underscore the need for practical household-level interventions, such as ring vaccination [34], or case-area targeted interventions (CATIs) used in cholera outbreaks, to curb intra-household transmission [35].

The PCR positivity rate in this study was higher among individuals aged ≥ 15 years. Similar findings were seen in Kamituga, where positivity was significantly higher in those aged ≥ 15 years compared with < 15 years (84.5% vs 66.2%; p < 0.001) [13]. Possible explanations include a likely higher burden of non-mpox rash illnesses (e.g. measles or varicella) in children attending MTCs, or differences in lesion type and location. Genital lesions, more common in older age groups, may carry higher viral loads [36]; if they are also more likely to be sampled (though we lack data on this), this could contribute to the higher PCR positivity rates in adults. Finally, samples were not collected systematically, mainly based on availability of materials. While we have no evidence of bias in sample collection, we cannot exclude the possibility that certain populations were preferentially sampled. Further investigations are needed to better understand the driver of differential MPXV PCR positivity by age.

Our findings underscore the urgent need for integrated mpox programmes targeting key vulnerable populations. The proportion of HIV-positivity among suspected mpox cases during this early phase of the outbreak in Uvira was 1.8%, higher than the estimated national average of 0.7% [37], and the 0.2% prevalence among voluntary blood donors at Uvira Hospital in 2024. Limited access to CD4 ^+^ T-cell count monitoring and opportunistic infection management complicates treatment, highlighting the importance of integrated mpox–HIV care models and vaccine safety studies for people with weakened immune systems. As not all patients were tested for HIV, and testing was influenced by clinical presentation, selection bias cannot be excluded; therefore, the proportions observed at the MTC may not reflect the prevalence in Uvira.

Among children, undernutrition is the leading cause of acquired immune deficiency and is especially concerning in the DRC where nearly half of children aged < 5 years are stunted [38]. Our study found acute malnutrition rates (12.1%) among suspected mpox cases nearly double the 2023–2024 South Kivu province average of 6.7% [38]. This figure is likely an underestimate, as we relied solely on MUAC measurements without weight-for-height z-scores [39]. Lastly, pregnant and breastfeeding cases, who represented 14.5% and 22.5% of females of reproductive age at the Uvira MTC, respectively, also require specific attention given the high CFR of MPXV clade Ib in neonates and emerging evidence suggesting potential mother-to-child transmission, including through breast milk [16]. Thus, our findings support WHO recommendations advocating for the vaccination of pregnant and breast-feeding females [40,41] and emphasise the need for greater investment in understanding the interplay between MPXV clade Ib, age, malnutrition, HIV and pregnancy. Addressing these gaps requires urgent attention, in-depth investigations and targeted interventions that extend beyond current emergency responses.

We observed one apparent mpox-related adverse outcome in pregnancy through home visits conducted after the main data collection window among pregnant females residing in Uvira. In Kamituga, a major mining hub, eight of 14 pregnant women with MPXV clade Ib reported abortion, and MPXV DNA was detected in one placenta [16]. Conflicting evidence on mother-to-child transmission of MPXV clade II further highlights existing knowledge gaps [5,42]. Differences in environmental exposures, mpox severity, mode of acquisition and co-infections, such as HIV or congenital co-infections, may contribute to the observed disparities. In South Kivu, birth defect rates were nearly four times higher in mining areas like Mwenga, near Kamituga, even before the mpox outbreak [43]. Research from Lubumbashi, over 1,000 km south, linked paternal mining exposure, prenatal metal levels, and poor maternal nutrition to malformations in mining sites [44]. Additionally, the level of commercial sex activity in Kamituga raises concerns about co-infections that may exacerbate pregnancy complications. Only one syphilis case was detected among 276 patients in Uvira, with no comparable data from Kamituga. Our limited data do not allow us to draw general conclusions on the impacts of mpox on pregnancy outcomes. Larger studies, including pooled analyses from multiple settings, are needed to clarify the potential causal relationship between MPXV clade Ib infection and adverse pregnancy outcomes.

This study has several limitations. It relied on facility-based surveillance from MTC due to the lack of data on cases in private facilities or the community. The clinical case definition used in the MTC was broad, and the low proportion of cases tested may have led to misclassification. However, the high positivity rate among those tested provides some assurance that case definition was generally adequate. Additionally, many patients were admitted to the MTC primarily for isolation, sometimes at their own request, rather than for inpatient care, limiting the interpretability of the impact of hospitalisation status in our dataset, particularly in the outbreak’s early months. The dataset also lacked precision regarding lesion distribution. For example, due to the structure of the questionnaire used in routine surveillance, we were unable to distinguish patients with isolated genital lesions from those with genital lesions in addition to lesions elsewhere on the body, limiting our ability to assess the role of sexual transmission in Uvira compared with other settings like Kamituga.

Our reliance on the GeneXpert; Xpert Mpox assay posed several challenges. While the high positivity rate supports the clinical case definition used, this test detects MPXV clade II and non-variola orthopoxviruses, but cannot directly confirm clade I. In our context, clade I was inferred when results were positive for orthopoxvirus and negative for clade II. Although this approach aligns with WHO guidance and is common in the DRC, its performance, especially for clade Ib, remains poorly characterised. However, a clade-typed subsample from Uvira confirmed all tested cases as clade Ib, providing some reassurance.

Operational constraints further limited diagnostic accuracy. These included the absence of on-site testing, delays in sample transport and processing which may have affected specimen quality, GeneXpert stockouts, competing demands from tuberculosis testing (which uses the same GeneXpert platforms), and communication gaps with laboratories. Testing for other common causes of skin lesions (e.g. varicella-zoster, measles, herpes simplex virus) was not routinely performed, making it difficult to determine the aetiology of mpox-negative cases. Misclassification, whether from clinical diagnosis or imperfect testing, not only affects burden estimation but also raises infection control concerns. Patients admitted to the MTCs based on clinical suspicion alone may have been exposed to, or may have exposed others to, infectious diseases. For this reason, we included both confirmed and unconfirmed cases to reflect the real-world complexity of mpox management and to highlight the urgent need for decentralised diagnostics, dedicated platforms, and stronger laboratory networks in outbreak-prone settings. To assess the influence of PCR-negative cases on the observed trends, we conducted a sensitivity analysis excluding patients who tested negative for MPXV. The overall clinical and epidemiologic trends remained consistent.

## Conclusion

The Uvira outbreak signalled a major shift in MPXV transmission, with household-driven spread disproportionately affecting children. This pattern, distinct from the clade II outbreaks in men who have sex with men and early clade Ib cases in Kamituga linked to heterosexual transmission, presents a global risk. Surveillance suggests similar patterns of non-sexual household transmission may be occurring throughout much of South Kivu, emphasising the need for broader interventions. Urgent global action including accelerating paediatric vaccine development, expanding therapeutic access, and implementing household-level interventions, is essential to contain clade Ib and mitigate its long-term health impacts on individuals and populations.

## Data Availability

De-identified participant data to generate the study findings are available from the corresponding author, upon reasonable request.

## Supplementary Data

534000 Supplementary Material
