# Supplementary material for: Community transmission of mpox clade Ib not driven through sexual exposures, Uvira, eastern Democratic Republic of the Congo, June to October 2024
Source: Euro Surveill. 2025 Dec 18;30(50):2500280. doi: 10.2807/1560-7917.ES.2025.30.50.2500280 (PMC12719941; doi:10.2807/1560-7917.ES.2025.30.50.2500280)
Supplement: Supplementary Material [file 25-00280_MALEMBAKA_Supplement.pdf]

Community transmission of mpox clade Ib not driven through sexual exposures, Uvira, eastern Democratic Republic of the Congo, June to October 2024

Patrick Musole Bugeme, Patrick Kazuba Bugale, Trust Faraja Mukika, Megan O'Driscoll, Javier Perez-Saez, Levi Bugwaja, Salomon Mashupe Shangula, Willy Kasi, Justin Bengehya, Stephanie Ngai, Antonio Isidro Carrion Martin, Jules Jackson, Patrick Katoto, Esto Bahizire, Noella Mulopo-Mukanya, Justin Lessler, Jackie Knee, Pauline Vetter, Elizabeth C Lee, Daniel Mukadi-Bamuleka, Andrew S Azman, Espoir Bwenge Malembaka

Supplementary document

This supplementary material is hosted by Eurosurveillance as supporting information alongside the article [Title], on behalf of the authors, who remain responsible for the accuracy and appropriateness of the content. The same standards for ethics, copyright, attributions and permissions as for the article apply. Supplements are not edited by Eurosurveillance and the journal is not responsible for the maintenance of any links or email addresses provided therein.

Content

1. Mpox case definition and description of patients 2
2. Mpox confirmation challenges in Uvira 4
3. Pregnancy status in the Mpox Treatment Center in Uvira 7
4. Characteristics and trends of mpox in Uvira among confirmed and untested cases 8

1. Mpox case definition and description of patients

The DRC's Ministry of Health, through the Programme National de Lutte contre le Monkeypox et les Fièvres Hémorragiques Virales (PNLMPX-FHV), considers a suspect mpox case as 1) anyone with a sudden high fever followed by a vesiculo-pustular rash predominantly on the face and present on the palms of the hands and soles of the feet; 2) or presence of at least 5 smallpox-type scars, 3) or anyone with fever > 38.3°C (101 F), severe headache, lymphadenopathy, back pain, myalgia, and severe asthenia, followed one to three days later by a progressive rash that often begins on the face (the densest) and then spreads elsewhere on the body, including the soles of the feet and palms of the hands. The community case definition of mpox is any person presenting with fever and skin rash. At the Uvira MTC, after the outbreak was confirmed, anyone presenting with a skin rash or lesions was considered a suspected mpox case and eligible for this study (Table S1).

Table S1. Socio-demographics characteristics and exposure history, by sex, among suspected mpox cases seeking healthcare at the Uvira Mpox Treatment Centre, Democratic Republic of the Congo, 3 June–24 October 2024 (n = 973)

Characteristic	Overall (n = 973)	Female (n = 491)	Male (n= 482)	p-value
Age in years, median (IQR)	9 (3.0, 20.0)	10 (4.0, 21.0)	8 (3.0, 18.0)	0.051
Age group				0.125
Under 1 year	101/973 (10.4%)	49/491 (10.0%)	52/482 (10.8%)	
1-4 years	211/973 (21.7%)	91/491 (18.5%)	120/482 (24.9%)	
5-14 years	308/973 (31.7%)	162/491 (33.0%)	146/482 (30.3%)	
15-44 years	327/973 (33.6%)	177/491 (36.0%)	150/482 (31.1%)	
≥ 45 years	26/973 (2.7%)	12/491 (2.4%)	14/482 (2.9%)	
Household size	8 (5.0, 10.0)	8 (5.0, 10.0)	8 (5.0, 10.0)	0.466
Number of children under 5 in the household				0.578
One to two	404/632 (63.9%)	204/320 (63.8%)	200/312 (64.1%)	
Three or more	106/632 (16.8%)	50/320 (15.6%)	56/312 (17.9%)	
Zero	122/632 (19.3%)	66/320 (20.6%)	56/312 (17.9%)	
Number of people per sleeping room in household, median (IQR)*				0.358
Median (Q1, Q3)	4 (2.5, 5.0)	4 (2.5, 5.0)	4 (2.7, 5.5)	
Student	310/973 (31.9%)	151/491 (30.8%)	159/482 (33.0%)	0.455
Pre-school age children	293/973 (30.1%)	134/491 (27.3%)	159/482 (33.0%)	0.053
Doing small business	83/973 (8.53%)	65/491 (13.2%)	18/482 (3.73%)	<0.001
Unemployed	69/973 (7.09%)	55/491 (11.2%)	14/482 (2.90%)	<0.001
Farmer	18/973 (1.85%)	13/491 (2.65%)	5/482 (1.04%)	0.062
Sex worker	12/973 (1.23%)	12/491 (2.44%)	0/482 (0%)	<0.001
Educator/Teacher	9/973 (0.92%)	3/491 (0.61%)	6/482 (1.24%)	0.337
Driver	12/973 (1.23%)	0/491 (0%)	12/482 (2.49%)	<0.001
Artisan (manual work)	9/973 (0.92%)	6/491 (1.22%)	3/482 (0.62%)	0.506
Cyclist	7/973 (0.72%)	0/491 (0%)	7/482 (1.45%)	0.007
Health care worker	4/973 (0.41%)	2/491 (0.41%)	2/482 (0.41%)	>0.999
Number of sexual partners in the past 3 weeks**				<0.001
Zero	84/286 (29.4%)	38/149 (25.5%)	46/137 (33.6%)	
One	153/286 (53.5%)	96/149 (64.4%)	57/137 (41.6%)	
2 to 5	42/286 (14.7%)	9/149 (6.04%)	33/137 (24.1%)	
At least 6	7/286 (2.45%)	6/149 (4.03%)	1/137 (0.73%)	
Had contact with a suspected mpox case***	346/743 (46.6%)	174/381 (45.7%)	172/362 (47.5%)	0.614
Number of contacts with a suspected mpox case***				0.087

One	276/346 (79.8%)	146/174 (83.9%)	130/172 (75.6%)	
Two	43/346 (12.4%)	15/174 (8.62%)	28/172 (16.3%)	
At least 3	27/346 (7.80%)	13/174 (7.47%)	14/172 (8.14%)	
Sexual exposure to a suspected mpox case**	19/318 (5.97%)	12/158 (7.59%)	7/160 (4.38%)	0.226
Working in mining site	4/973 (0.41%)	1/491 (0.20%)	3/482 (0.62%)	0.370
Had contact with animal	39/776 (5.03%)	17/395 (4.30%)	22/381 (5.77%)	0.349
Type of animal contact				0.436
Domestic animals	38/39 (97.4%)	16/17 (94.1%)	22/22 (100.0%)	
Wild animals	1/39 (2.56%)	1/17 (5.88%)	0/22 (0%)	
Traveled outside Uvira***	90/845 (10.7%)	40/426 (9.39%)	50/419 (11.9%)	0.231
International travel***				0.419
Burundi	30/31 (96.8%)	12/13 (92.3%)	18/18 (100.0%)	
Tanzania	1/31 (3.23%)	1/13 (7.69%)	0/18 (0%)	

Data are median (IQR) or n/N (%), N representing the number of individuals with non-missing data. *Household characteristics data were not available for non-residents of Uvira city and Uvira residents for whom a household visit was not conducted mainly due lack of accurate residential address. **Sexual exposure was reported only for individuals aged ≥ 15 years. Occupation was a multiple-choice question. ***All questions about exposure and travel history referred to the three weeks preceding the onset of fever or skin rash.

Table S2. Clinical characteristics of suspected mpox cases, by sex, on admission to Uvira Mpox Treatment Centre, the Democratic Republic of Congo, 3 June–24 October 2024 (n = 973)

**Characteristic**	**Overall** (n = 973)	**Female** (n= 491)	**Male** (n= 482)	**p-value**
Hospitalized	431/860 (50.1%)	225/434 (51.8%)	206/426 (48.4%)	0.307
Self-reported fever	648/836 (77.5%)	326/425 (76.7%)	322/411 (78.3%)	0.570
Lesions on palms and soles	320/872 (36.7%)	152/443 (34.3%)	168/429 (39.2%)	0.137
Cervical adenopathy	550/870 (63.2%)	267/439 (60.8%)	283/431 (65.7%)	0.139
Inguinal adenopathy	664/872 (76.1%)	325/442 (73.5%)	339/430 (78.8%)	0.066
Axillary adenopathy	288/867 (33.2%)	137/439 (31.2%)	151/428 (35.3%)	0.203
Generalized adenopathy	205/840 (24.4%)	101/425 (23.8%)	104/415 (25.1%)	0.662
Eye redness	113/861 (13.1%)	58/437 (13.3%)	55/424 (13.0%)	0.896
Eye itching	74/513 (14.4%)	42/271 (15.5%)	32/242 (13.2%)	0.464
Light sensitivity	84/489 (17.2%)	46/261 (17.6%)	38/228 (16.7%)	0.779
Oral lesions	204/852 (23.9%)	104/428 (24.3%)	100/424 (23.6%)	0.807
Genital lesions	413/860 (48.0%)	193/433 (44.6%)	220/427 (51.5%)	0.041
Anal lesions	123/852 (14.4%)	60/431 (13.9%)	63/421 (15.0%)	0.665
Number of lesions				0.266
<25 lesions	238/858 (27.7%)	131/434 (30.2%)	107/424 (25.2%)	
25-99 lesions	429/858 (50.0%)	212/434 (48.8%)	217/424 (51.2%)	
100-250 lesions	125/858 (14.6%)	56/434 (12.9%)	69/424 (16.3%)	
>250 lesions	66/858 (7.69%)	35/434 (8.06%)	31/424 (7.31%)	
Pregnancy status*	19/131 (14.5%)	19/131 (14.5%)	-	-
Breastfeeding mother*	27/120 (22.5%)	27/120 (22.5%)	-	-
Breastfed child**	128/166 (77.1%)	67/80 (83.8%)	61/86 (70.9%)	0.049
Nutritional status of children				0.166
Severe acute malnutrition	10/181 (5.52%)	2/83 (2.41%)	8/98 (8.16%)	
Moderate acute malnutrition	12/181 (6.63%)	7/83 (8.43%)	5/98 (5.10%)	
Normal	159/181 (87.8%)	74/83 (89.2%)	85/98 (86.7%)	
Syphilis test				>0.999
Negative	272/273 (99.6%)	149/150 (99.3%)	123/123 (100.0%)	
Positive	1/273 (0.37%)	1/150 (0.67%)	0/123 (0%)	
HIV test				>0.999
Negative	323/329 (98.2%)	174/177 (98.3%)	149/152 (98.0%)	
Positive	6/329 (1.82%)	3/177 (1.69%)	3/152 (1.97%)	

Mpox confirmation (PCR)				0.992
Negative	93/415 (22.4%)	45/201 (22.4%)	48/214 (22.4%)	
Positive	322/415 (77.6%)	156/201 (77.6%)	166/214 (77.6%)	

*Questions about breastfeeding and pregnancy were only asked to female patients aged 15-49 years. **Child's breastfeeding status was only asked for those aged ≤ 2 years.

2. Mpox confirmation challenges in Uvira

During the study period, on-site confirmation of mpox was unavailable. Skin and/or oropharyngeal swabs were stored at 2–8°C in a refrigerator at the Uvira Health Zone office, often for 1–3 weeks or longer, before being transported to the laboratory in Bukavu, 120 km away (a 4–6-hour drive). Testing at the Bukavu lab was conducted using GeneXpert® platform (Xpert® Mpox, Cepheid, Sunnyvale, the United States (US)), on two 4-module machines used for tuberculosis testing.

In the early phases of the outbreak, all mpox samples from South Kivu, except those from the remote area of Kamituga, which had its own GeneXpert testing capacity, were sent to the Rodolphe Mérieux laboratory at the Institut National de Recherche Biomédicale (INRB) in Goma, North Kivu, for PCR confirmation. In June 2024, testing was decentralized. INRB Goma, in collaboration with the South Kivu health division and WHO, trained staff at the South Kivu laboratory and installed mpox GeneXpert modules on the existing tuberculosis machines. This enabled the South Kivu laboratory to handle samples from 33 of the province's 34 health zones (including Uvira). However, logistical and operational challenges persisted, including frequent stockouts of sample collection materials and cartridges, logistical delays in sample transportation, and processing backlogs. These delays may have affected specimen quality, although the extent to which they impact viral DNA integrity in nasopharyngeal or skin swabs specimens is not yet fully understood. During periods of stockouts in Bukavu, samples were rerouted to INRB Goma for testing.

The Bukavu laboratory, responsible for most of South Kivu, faced additional strain due to the dual use of machines for both mpox and tuberculosis testing, leading to breakdowns and delays in processing, and result interpretation. Communication challenges between the reference labs in Bukavu or Goma and the Uvira MTC further compounded delays, with results sometimes arriving weeks after patients had been discharged. Additionally, used cartridges were stored at the Bukavu laboratory until transported to Uvira, the only site equipped with an incinerator capable of safely destroying them. Figure S1 below shows the weekly testing rate for samples from Uvira during the study period.

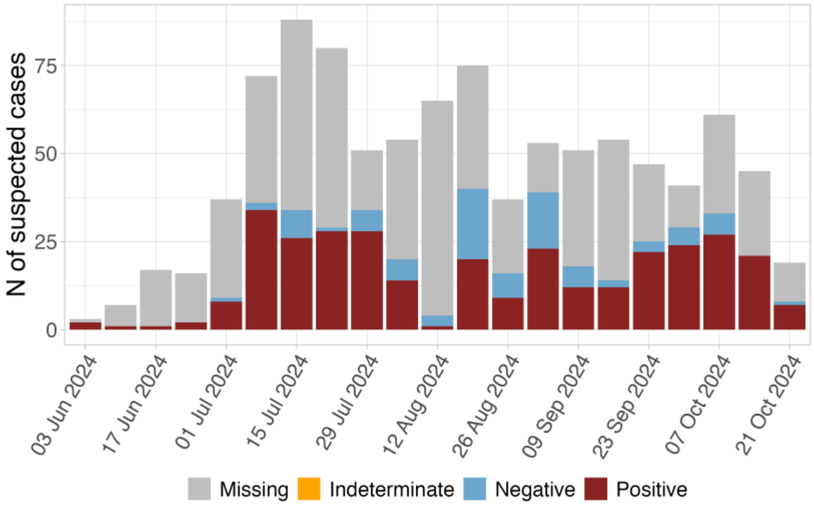

Figure S1. Epidemic curve of suspected mpox patients in Uvira by GeneXpert confirmation and week

A detailed comparison of tested and untested cases is presented in **Table S3**, while **Table S4** provides a comparison of positive and negative cases and **Table S5** summarises positive cases.

Table S3. Sociodemographic and clinical characteristics of laboratory-tested suspected mpox cases

**Characteristic**	**Overall** (n = 973)	**Tested** (n = 415)	**Untested** (n= 558)	**p-value**
Sex				0.275
Female	491/973 (50.5%)	201/415 (48.4%)	290/558 (52.0%)	
Male	482/973 (49.5%)	214/415 (51.6%)	268/558 (48.0%)	
Age in years, median (IQR)	9 (3.0, 20.0)	9 (2.8, 21.0)	9 (4.0, 19.0)	0.305
Age group				0.020
Under 1 year	101/973 (10.4%)	55/415 (13.3%)	46/558 (8.2%)	
1-4 years	211/973 (21.7%)	88/415 (21.2%)	123/558 (22.0%)	
5-14 years	308/973 (31.7%)	121/415 (29.2%)	187/558 (33.5%)	
15-44 years	327/973 (33.6%)	145/415 (34.9%)	182/558 (32.6%)	
≥ 45 years	26/973 (2.7%)	6/415 (1.4%)	20/558 (3.6%)	
Hospitalized	431/860 (50.1%)	238/395 (60.3%)	193/465 (41.5%)	<0.001
Self-reported fever	648/836 (77.5%)	322/388 (83.0%)	326/448 (72.8%)	<0.001
Lesions on palms and soles	320/872 (36.7%)	172/397 (43.3%)	148/475 (31.2%)	<0.001
Cervical adenopathy	550/870 (63.2%)	289/399 (72.4%)	261/471 (55.4%)	<0.001
Inguinal adenopathy	664/872 (76.1%)	340/399 (85.2%)	324/473 (68.5%)	<0.001
Axillary adenopathy	288/867 (33.2%)	171/398 (43.0%)	117/469 (24.9%)	<0.001
Generalized adenopathy	205/840 (24.4%)	131/382 (34.3%)	74/458 (16.2%)	<0.001
Eye redness	113/861 (13.1%)	62/394 (15.7%)	51/467 (10.9%)	0.037
Eye itching	74/513 (14.4%)	45/227 (19.8%)	29/286 (10.1%)	0.002
Light sensitivity	84/489 (17.2%)	50/218 (22.9%)	34/271 (12.5%)	0.002
Oral lesions	204/852 (23.9%)	125/392 (31.9%)	79/460 (17.2%)	<0.001
Genital lesions	413/860 (48.0%)	221/396 (55.8%)	192/464 (41.4%)	<0.001
Anal lesions	123/852 (14.4%)	62/392 (15.8%)	61/460 (13.3%)	0.290
Number of lesions				0.121
<25 lesions	238/858 (27.7%)	99/395 (25.1%)	139/463 (30.0%)	
25-99 lesions	429/858 (50.0%)	200/395 (50.6%)	229/463 (49.5%)	
100-250 lesions	125/858 (14.6%)	68/395 (17.2%)	57/463 (12.3%)	
>250 lesions	66/858 (7.69%)	28/395 (7.09%)	38/463 (8.21%)	
Pregnancy status	19/131 (14.5%)	8/61 (13.1%)	11/70 (15.7%)	0.673
Nutritional status of children				0.484
Severe acute malnutrition	10/181 (5.52%)	4/96 (4.17%)	6/85 (7.06%)	
Moderate acute malnutrition	12/181 (6.63%)	5/96 (5.21%)	7/85 (8.24%)	

Normal	159/181 (87.8%)	87/96 (90.6%)	72/85 (84.7%)	
Syphilis test result				>0.999
Negative	272/273 (99.6%)	125/125 (100.0%)	147/148 (99.3%)	
Positive	1/273 (0.37%)	0/125 (0%)	1/148 (0.68%)	
HIV test result				0.448
Negative	323/329 (98.2%)	160/164 (97.6%)	163/165 (98.8%)	
Positive	6/329 (1.82%)	4/164 (2.44%)	2/165 (1.21%)	

Table S4. Description of suspected mpox cases by PCR result

**Characteristic**	**Overall** (n = 415)	**Negative** (n = 93)	**Positive** (n = 322)	**p-value**
Sex				0.992
Female	201/415 (48.4%)	45/93 (48.4%)	156/322 (48.4%)	
Male	214/415 (51.6%)	48/93 (51.6%)	166/322 (51.6%)	
Age in years, median (IQR)	9 (2.8, 21.0)	7 (2.0, 16.0)	10 (3.0, 22.0)	0.134
Age group				0.131
Under 1 year	55/415 (13.3%)	12/93 (12.9%)	43/322 (13.4%)	
1-4 years	88/415 (21.2%)	23/93 (24.7%)	65/322 (20.2%)	
5-14 years	121/415 (29.2%)	33/93 (35.5%)	88/322 (27.3%)	
15-44 years	145/415 (34.9%)	23/93 (24.7%)	122/322 (37.9%)	
≥ 45 years	6/415 (1.4%)	2/93 (2.2%)	4/322 (1.2%)	
Hospitalized	238/395 (60.3%)	39/85 (45.9%)	199/310 (64.2%)	0.002
Self-reported fever	322/388 (83.0%)	67/83 (80.7%)	255/305 (83.6%)	0.535
Lesions on palms and soles	172/397 (43.3%)	27/87 (31.0%)	145/310 (46.8%)	0.009
Cervical adenopathy	289/399 (72.4%)	57/86 (66.3%)	232/313 (74.1%)	0.149
Inguinal adenopathy	340/399 (85.2%)	75/86 (87.2%)	265/313 (84.7%)	0.556
Axillary adenopathy	171/398 (43.0%)	27/86 (31.4%)	144/312 (46.2%)	0.014
Generalized adenopathy	131/382 (34.3%)	20/82 (24.4%)	111/300 (37.0%)	0.033
Eye redness	62/394 (15.7%)	10/85 (11.8%)	52/309 (16.8%)	0.256
Eye itching	45/227 (19.8%)	7/47 (14.9%)	38/180 (21.1%)	0.341
Light sensitivity	50/218 (22.9%)	7/45 (15.6%)	43/173 (24.9%)	0.186
Oral lesions	125/392 (31.9%)	16/87 (18.4%)	109/305 (35.7%)	0.002
Genital lesions	221/396 (55.8%)	45/86 (52.3%)	176/310 (56.8%)	0.462
Anal lesions	62/392 (15.8%)	8/86 (9.30%)	54/306 (17.6%)	0.061
Number of lesions				0.400
<25 lesions	99/395 (25.1%)	25/85 (29.4%)	74/310 (23.9%)	
25-99 lesions	200/395 (50.6%)	45/85 (52.9%)	155/310 (50.0%)	
100-250 lesions	68/395 (17.2%)	11/85 (12.9%)	57/310 (18.4%)	
>250 lesions	28/395 (7.09%)	4/85 (4.71%)	24/310 (7.74%)	
Pregnancy status	8/61 (13.1%)	2/10 (20.0%)	6/51 (11.8%)	0.607
Nutritional status of children				0.488
Severe acute malnutrition	4/96 (4.17%)	0/24 (0%)	4/72 (5.56%)	
Moderate acute malnutrition	5/96 (5.21%)	2/24 (8.33%)	3/72 (4.17%)	
Normal	87/96 (90.6%)	22/24 (91.7%)	65/72 (90.3%)	
Syphilis test result				
negative	125/125 (100.0%)	28/28 (100.0%)	97/97 (100.0%)	
HIV test result				0.577
Negative	160/164 (97.6%)	46/46 (100.0%)	114/118 (96.6%)	
Positive	4/164 (2.44%)	0/46 (0%)	4/118 (3.39%)	

Table S5. Description of confirmed mpox cases by age

Characteristic	Overall N = 322	Under 15 years N = 196	≥15 years N = 126	p-value
Sex				0.112
female	156/322 (48.4%)	88/196 (44.9%)	68/126 (54.0%)	
male	166/322 (51.6%)	108/196 (55.1%)	58/126 (46.0%)	
Hospitalized	199/310 (64.2%)	121/192 (63.0%)	78/118 (66.1%)	0.583
Self-reported fever	255/305 (83.6%)	161/189 (85.2%)	94/116 (81.0%)	0.342
Lesions on palms and soles	145/310 (46.8%)	93/193 (48.2%)	52/117 (44.4%)	0.522
Cervical adenopathy	232/313 (74.1%)	159/194 (82.0%)	73/119 (61.3%)	<0.001
Inguinal adenopathy	265/313 (84.7%)	163/194 (84.0%)	102/119 (85.7%)	0.686
Axillary adenopathy	144/312 (46.2%)	104/193 (53.9%)	40/119 (33.6%)	<0.001
Generalized adenopathy	111/300 (37.0%)	83/184 (45.1%)	28/116 (24.1%)	<0.001
Eye redness	52/309 (16.8%)	28/191 (14.7%)	24/118 (20.3%)	0.195
Eye itching	38/180 (21.1%)	17/63 (27.0%)	21/117 (17.9%)	0.157
Light sensitivity	43/173 (24.9%)	17/58 (29.3%)	26/115 (22.6%)	0.336
Oral lesions	109/305 (35.7%)	67/188 (35.6%)	42/117 (35.9%)	0.963
Genital lesions	176/310 (56.8%)	88/193 (45.6%)	88/117 (75.2%)	<0.001
Anal lesions	54/306 (17.6%)	34/190 (17.9%)	20/116 (17.2%)	0.884
Number of lesions				0.002
<25 lesions	74/310 (23.9%)	34/193 (17.6%)	40/117 (34.2%)	
25-99 lesions	155/310 (50.0%)	98/193 (50.8%)	57/117 (48.7%)	
100-250 lesions	57/310 (18.4%)	45/193 (23.3%)	12/117 (10.3%)	
>250 lesions	24/310 (7.7%)	16/193 (8.3%)	8/117 (6.8%)	
HIV test result				0.028
Negative	114/118 (96.6%)	69/69 (100.0%)	45/49 (91.8%)	
Positive	4/118 (3.4%)	0/69 (0.0%)	4/49 (8.2%)	

3. Pregnancy status in the Mpox Treatment Center in Uvira

Between June 3 and October 24, 2024, we recorded 131 women of childbearing age (15-49 years), of whom 19 self-reported to be pregnant (Table S6). No pregnancy test was offered in the MTC at the time of this study.

Table S6. Description pregnant women in the Uvira Mpox Treatment Center

Characteristic	N = 19
Age	21 (19.0, 28.0)
Gestational age	
Q1	5/19 (26.3%)
Q2	5/19 (26.3%)
Q3	8/19 (42.1%)
Unknown gestational age	1/19 (5.3%)
Hospitalized	13/19 (68.4%)
Self-reported fever	13/19 (68.4%)
Presence of genital lesions	12/19 (63.2%)
Presence of anal lesions	3/19 (15.8%)
Number of lesions	

<25 lesions	7/19 (36.8%)
25-99 lesions	9/19 (47.4%)
100-250 lesions	1/19 (5.26%)
>250 lesions	2/19 (10.5%)
Absence of vaginal bleeding	17/17 (100.0%)
Absence of uterine contractions	16/16 (100.0%)
Negative syphilis test result	6/6 (100.0%)
HIV test results	
Negative	6/7 (85.7%)
Positive	1/7 (14.3%)
Mpox confirmation (PCR)	
Positive	6/8 (75.0%)
Negative	1/8 (12.5%)
Indeterminate	1/8 (12.5%)

Variations in the denominator reflect differences in data availability for each characteristic.

4. Characteristics and trends of mpox in Uvira among confirmed and untested cases

To examine the impact of PCR-negative cases on the observed trends, we repeated the main analysis excluding patients who tested negative for mpox (n = 93), retaining only PCR-positive and untested cases. The overall trends remained consistent.

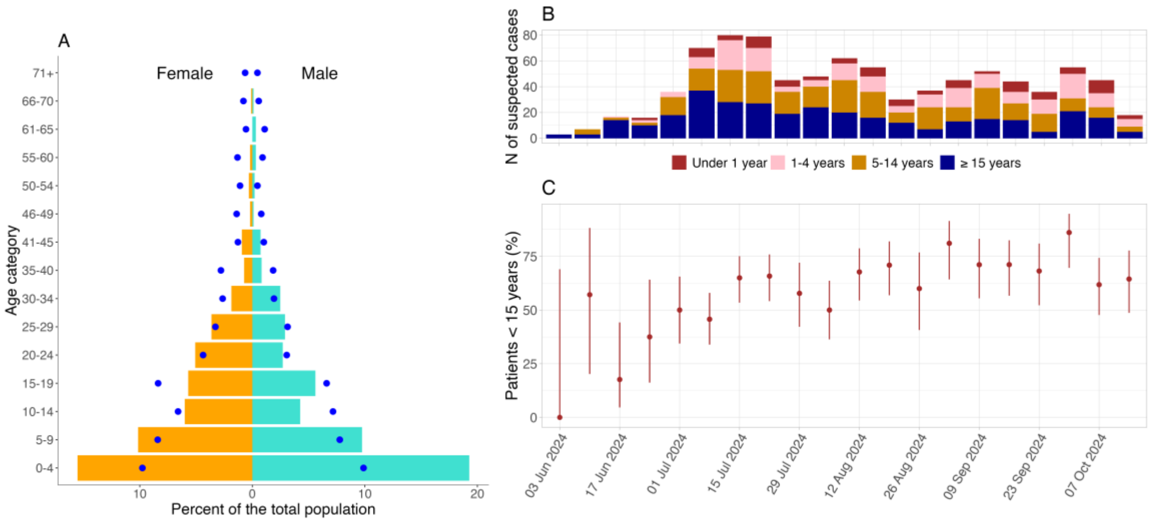

Figure S2. Description of the mpox epidemic in Uvira, excluding PCR-negative cases. Panel A: Age-sex pyramid of suspected cases and the general population of Uvira, with the bars representing the proportion of men (turquoise) and women (orange) by age group within the study population. The blue dots denote the disaggregation by age and sex from the population estimates based on household composition data from four cross-sectional surveys conducted in Uvira between 2021-2024 (n = 10,604 household members). Panel B: Epidemic curve by age group and week of presentation at the Mpox Treatment Center (MTC). Panel C: Weekly proportion of children under 15 years of age presenting to the MTC.

Table S7. Socio-demographic characteristics and exposure history of suspected mpox cases seeking care at the Uvira Mpox Treatment Centre, excluding PCR-negative cases

Characteristic	Overall N = 880	Under 15 years N = 552	≥15 years N = 328	p-value
Age in years, median (IQR)				
Median (Q1, Q3)	9 (3.0, 20.0)	4 (1.9, 8.0)	24 (19.0, 31.0)	
Sex of the patient				0.173
female	446/880 (50.7%)	270/552 (48.9%)	176/328 (53.7%)	
male	434/880 (49.3%)	282/552 (51.1%)	152/328 (46.3%)	
Household size, median (IQR)*				0.116
Median (Q1, Q3)	8 (5.0, 10.0)	8 (6.0, 10.0)	7 (5.0, 10.0)	
Number of people per sleeping room in household, median (IQR)*	4 (2.5, 5.0)	4 (3.0, 5.0)	3 (2.0, 5.0)	<0.001
Occupation				
Student	276/880 (31.4%)	219/552 (39.7%)	57/328 (17.4%)	NA
Pre-school age children	256/880 (29.1%)	256/552 (46.4%)	0/328 (0.0%)	NA
Doing small business	79/880 (9.0%)	7/552 (1.3%)	72/328 (22.0%)	NA
Farmer	18/880 (2.0%)	6/552 (1.1%)	12/328 (3.7%)	NA
Sex worker	11/880 (1.3%)	0/552 (0.0%)	11/328 (3.4%)	NA
Educator/Teacher	9/880 (1.0%)	0/552 (0.0%)	9/328 (2.7%)	NA
Driver	11/880 (1.3%)	0/552 (0.0%)	11/328 (3.4%)	NA
Artisan (manual work)	7/880 (0.8%)	1/552 (0.2%)	6/328 (1.8%)	NA
Cyclist	7/880 (0.8%)	0/552 (0.0%)	7/328 (2.1%)	NA
Health care worker	4/880 (0.5%)	0/552 (0.0%)	4/328 (1.2%)	NA
Working in mining site	4/880 (0.5%)	2/552 (0.4%)	2/328 (0.6%)	NA
No occupation	65/880 (7.4%)	6/552 (1.1%)	59/328 (18.0%)	NA
Number of sexual partners in the past 3 weeks**				
Zero	75/263 (28.5%)	NA	75/263 (28.5%)	
One	143/263 (54.4%)	NA	143/263 (54.4%)	
2 to 5	39/263 (14.8%)	NA	39/263 (14.8%)	
At least 6	6/263 (2.3%)	NA	6/263 (2.3%)	
Had contact with a suspected mpox case***	305/661 (46.1%)	207/416 (49.8%)	98/245 (40.0%)	0.015
Number of contacts with a suspected mpox case***				0.096
One	239/305 (78.4%)	156/207 (75.4%)	83/98 (84.7%)	
Two	40/305 (13.1%)	33/207 (15.9%)	7/98 (7.1%)	
At least 3	26/305 (8.5%)	18/207 (8.7%)	8/98 (8.2%)	
Sexual exposure to a suspected mpox case**	16/282 (5.7%)	0/190 (0.0%)	16/92 (17.4%)	<0.001
Had contact with animal	33/692 (4.8%)	22/446 (4.9%)	11/246 (4.5%)	0.785
Type of animal contact				0.333
Domestic animals	32/33 (97.0%)	22/22 (100.0%)	10/11 (90.9%)	
Wild animals	1/33 (3.0%)	0/22 (0.0%)	1/11 (9.1%)	
Traveled outside Uvira***	84/759 (11.1%)	16/484 (3.3%)	68/275 (24.7%)	<0.001
International travel***				>0.999
Burundi	26/27 (96.3%)	2/2 (100.0%)	24/25 (96.0%)	
Tanzania	1/27 (3.7%)	0/2 (0.0%)	1/25 (4.0%)	

Data are median (IQR) or n/N (%), N representing the number of individuals with non-missing data. *Household characteristics data were not available for non-residents of Uvira city and Uvira residents for whom a household visit was not conducted mainly due to lack of accurate residential address. **Sexual exposure was reported only for individuals aged ≥ 15 years who were considered likely to be sexually active. Occupation was a multiple-choice question. ***All questions about exposure and travel history referred to the three weeks preceding the onset of fever or skin rash.

Table S8: Description of clinical characteristics of suspected cases on admission, by age, excluding PCR-negative cases

Characteristic	Overall N = 880	Under 15 years N = 552	≥15 years N = 328	p-value
Sex				0.173
female	446/880 (50.7%)	270/552 (48.9%)	176/328 (53.7%)	
male	434/880 (49.3%)	282/552 (51.1%)	152/328 (46.3%)	
Hospitalized	392/775 (50.6%)	242/492 (49.2%)	150/283 (53.0%)	0.306
Self-reported fever	581/753 (77.2%)	375/475 (78.9%)	206/278 (74.1%)	0.126
Lesions on palms and soles	293/785 (37.3%)	179/498 (35.9%)	114/287 (39.7%)	0.292
Cervical adenopathy	493/784 (62.9%)	354/497 (71.2%)	139/287 (48.4%)	<0.001
Inguinal adenopathy	589/786 (74.9%)	365/499 (73.1%)	224/287 (78.0%)	0.127
Axillary adenopathy	261/781 (33.4%)	189/495 (38.2%)	72/286 (25.2%)	<0.001
Generalized adenopathy	185/758 (24.4%)	142/479 (29.6%)	43/279 (15.4%)	<0.001
Eye redness	103/776 (13.3%)	62/492 (12.6%)	41/284 (14.4%)	0.468
Eye itching	67/466 (14.4%)	31/185 (16.8%)	36/281 (12.8%)	0.235
Light sensitivity	77/444 (17.3%)	33/170 (19.4%)	44/274 (16.1%)	0.364
Oral lesions	188/765 (24.6%)	121/486 (24.9%)	67/279 (24.0%)	0.785
Genital lesions	368/774 (47.5%)	189/492 (38.4%)	179/282 (63.5%)	<0.001
Anal lesions	115/766 (15.0%)	68/486 (14.0%)	47/280 (16.8%)	0.297
Number of lesions				<0.001
<25 lesions	213/773 (27.6%)	109/492 (22.2%)	104/281 (37.0%)	
25-99 lesions	384/773 (49.7%)	253/492 (51.4%)	131/281 (46.6%)	
100-250 lesions	114/773 (14.7%)	92/492 (18.7%)	22/281 (7.8%)	
>250 lesions	62/773 (8.0%)	38/492 (7.7%)	24/281 (8.5%)	
Pregnancy status	17/121 (14.0%)	NA	17/121 (14.0%)	NA
Breastfeeding mother	24/109 (22.0%)	NA	24/109 (22.0%)	NA
Breastfed child	108/142 (76.1%)	108/142 (76.1%)	0NA	NA
Nutritional status of children 3 to 59 months old				NA
Severe acute malnutrition	10/157 (6.4%)	10/157 (6.4%)	NA	
Moderate acute malnutrition	10/157 (6.4%)	10/157 (6.4%)	NA	
Normal	137/157 (87.3%)	137/157 (87.3%)	0/0 (NA%)	
Syphilis test result				0.392
negative	244/245 (99.6%)	149/149 (100.0%)	95/96 (99.0%)	
positive	1/245 (0.4%)	0/149 (0.0%)	1/96 (1.0%)	
HIV test result				0.003
Negative	277/283 (97.9%)	177/177 (100.0%)	100/106 (94.3%)	
Positive	6/283 (2.1%)	0/177 (0.0%)	6/106 (5.7%)	
Mpox confirmation (PCR)				
Positive	322/322 (100.0%)	196/196 (100.0%)	126/126 (100.0%)	

*Child's breastfeeding status was only asked for those aged ≤ 2 years. **Questions on breastfeeding and pregnancy were only asked to female patients aged 15-49 years.

Table S9. Description of exposure location, relationships with suspected Mpox cases, and contact characteristics, excluding negative cases

Characteristic	Overall<br>N = 414	Under 15 years<br>N = 289	≥15 years<br>N = 125	p-value
Sex				<0.001
Male	214/414 (51.7%)	169/289 (58.5%)	45/125 (36.0%)	
Female	200/414 (48.3%)	120/289 (41.5%)	80/125 (64.0%)	
Number of contacts				0.003
Multiple	337/359 (93.9%)	239/248 (96.4%)	98/111 (88.3%)	
Once	22/359 (6.1%)	9/248 (3.6%)	13/111 (11.7%)	
Relationship with the suspected case				
Child	29/378 (7.7%)	0/261 (0.0%)	29/117 (24.8%)	
Parent	11/378 (2.9%)	9/261 (3.4%)	2/117 (1.7%)	
Spouse	10/378 (2.6%)	0/261 (0.0%)	10/117 (8.5%)	
Health care exposure	6/378 (1.6%)	3/261 (1.1%)	3/117 (2.6%)	
Sex partner	6/378 (1.6%)	0/261 (0.0%)	6/117 (5.1%)	
Another household member (e.g. sibling)	184/378 (48.7%)	153/261 (58.6%)	31/117 (26.5%)	
Another non-household relative, friend, colleague, neighbor	132/378 (34.9%)	96/261 (36.8%)	36/117 (30.8%)	
Contact happened in Uvira	379/393 (96.4%)	267/272 (98.2%)	112/121 (92.6%)	0.014
Reported contact person was hospitalized	165/201 (82.1%)	124/147 (84.4%)	41/54 (75.9%)	0.167
Exposure at bars, hotels, night clubs	93/393 (23.7%)	63/272 (23.2%)	30/121 (24.8%)	0.725
Household exposure	272/393 (69.2%)	191/272 (70.2%)	81/121 (66.9%)	0.516
Exposure at workplace	5/393 (1.3%)	0/272 (0.0%)	5/121 (4.1%)	0.003
Exposure at school	3/393 (0.8%)	3/272 (1.1%)	0/121 (0.0%)	0.556
Exposure at sport place	1/393 (0.3%)	1/272 (0.4%)	0/121 (0.0%)	>0.999
Exposure at health facility	13/393 (3.3%)	5/272 (1.8%)	8/121 (6.6%)	0.027

All questions refer to the 3 weeks before symptom onset.
